# The acceptance and hesitancy of COVID-19 vaccination among chronic obstructive pulmonary disease (COPD) patients

**DOI:** 10.1097/MD.0000000000033923

**Published:** 2023-06-30

**Authors:** Ting Wang, Yang Bai, Lele Bai, Ning Wang

**Affiliations:** a Department of Respiratory Medicine, Xi’an People’s Hospital (Xi’an No.4 Hospital), Xi’an, China; b Department of Medical Ultrasonics, Xi’an People’s Hospital (Xi’an No.4 Hospital), Xi’an, China; c Department of General Practice, Xi’an People’s Hospital (Xi’an No.4 Hospital), Xi’an, China.

**Keywords:** acceptance, COPD, COVID-19, hesitancy, vaccine

## Abstract

Severe acute respiratory syndrome coronavirus 2, which is responsible for the coronavirus disease 2019 (COVID-19), causes severe clinical outcomes in old individuals and patients with underlying diseases, including chronic obstructive pulmonary disease (COPD). Considering vaccination is still the most effective method to prevent COVID-19-associated death, it is imperative to evaluate COPD patients’ attitudes toward the COVID-19 vaccine. This cross-sectional design study was conducted to assess vaccine acceptance and hesitancy among 212 COPD patients who attended the outpatient department from January 1, 2021, to July 31, 2022. All of the patients were not vaccinated and had undertaken lung function test at the time of our survey. Of 212 participants, 164 (77.4%) were willing to be vaccinated immediately while 48 (22.6%) were hesitant to be vaccinated. Compared with the acceptance group, patients who did not accept the vaccination instantly tended to have more comorbidities, like hypertension, coronary heart disease, recent cancers, and higher Modified British Medical Research Council score, or more frequent acute exacerbation. For the patients willing to be vaccinated, the main factors motivating them were an authorities-endorsed vaccine, free vaccination, and no obvious adverse reactions. For the hesitant group, no recommendation from the treating physician was the biggest obstacle for them to accept vaccination. Our results provide useful guidance for making intervention measures to enhance COPD patients’ acceptance of a new COVID-19 vaccine. For those patients with comorbidities, treating physicians promoting messages framing the safety of vaccination is necessary to increase immunization rates.

## 1. Introduction

Coronavirus disease 2019 (COVID-19) is a respiratory disease of respiratory infections that is mainly caused by severe acute respiratory syndrome coronavirus 2 (SARS-CoV-2). According to World Health Organization (WHO) coronavirus disease situation reports, COVID-19 has caused 687,600,227 infected cases and 6869,821 deaths worldwide as of May 6, 2023.^[1]^ Variously mild symptoms, such as fever, cough, anosmia, myalgia, fatigue, gastrointestinal disturbance, and sputum production are the common clinical manifestations of COVID-19.^[2]^ Moreover, SARS-CoV-2 sometimes leads to asymptomatic infection or severe pulmonary infection characterized by high fever, shortness of breath, or even respiratory and circulatory failure.^[3,4]^ All age groups were confirmed to be easily affected by SARS-CoV-2, but the population over 60 years, or individuals with basic diseases such as chronic obstructive pulmonary disease (COPD), asthma, diabetes, cardiovascular diseases (CVD), have a higher risk of developing infection and a worse prognosis.^[5]^ Even with the approval of some antiviral drugs, like remdesivir, bamlanivimab, and casirivimab, COVID-19 vaccine with sufficient efficacy and safety remains the top priority for not only preventing its spread but also restoring economic and social activities.^[6,7]^ From January 2021, COVID-19 vaccination was becoming available and the vast majority of people could access the vaccine on their request.^[8]^

Since COPD has high prevalence, morbidity, and mortality,^[9]^ there is a trend of growing cases suffering from COPD and COVID-19 at the same time along with COVID-19 pandemic. Thus, evaluating the COPD patients’ attitudes toward COVID-19 vaccine and developing measures to improve vaccination rates in these special populations has important significance for both clinical workers and authorities.

## 2. Materials and methods

### 2.1. Study participants

From January 1, 2021, to July 31, 2022, we recruited 212 diagnosed cases of COPD admitted to the respiratory medicine department of Xi’an People’s Hospital (Xi’an No.4 Hospital). All of the participants had not been vaccinated against COVID-19 and finished the lung function test. Each respondent was informed of the intention and every procedure of our study, signed a consent, then completed 2 standardized questionnaires that included general information regarding their age, marital status, present and past medical histories, as well as their attitudes toward COVID-19 vaccine. Our study, with the protection of privacy and no harm to the rights of patients, was conducted according to the principles of the Declaration of Helsinki. Informed consent was obtained from all the participants.

### 2.2. Clinical definition

The classification of airflow limitation severity in COPD is defined as follows: Global Initiative for Chronic Obstructive Lung Disease (GOLD) 1 (Mild), Forced Expiratory Volume in the first second (FEV1)/predicted ≥ 80%; GOLD 2 (Moderate), 50% ≤ FEV1 < 80% predicted; GOLD 3 (Severe), 30% ≤ FEV1 < 50% predicted; GOLD 4 (Very Severe), FEV1 < 30% predicted. We assessed every participant’s symptom using Modified British Medical Research Council (mMRC) Questionnaire (scale 1–5). It consists of 5 items and its value is set in a range from 0 (getting breathless with strenuous exercise) to 4 (too breathless to leave the house).^[10]^ Subsequently, Groups A to D were used to provide information regarding symptoms and exacerbation history. Specifically, group A patients were those with lower mMRC scores (0–1) and low risk of exacerbation (0 or 1, not leading to hospital admission). While, group B patients were those with low risk of exacerbation (0 or 1, not leading to hospital admission) but higher mMRC score (≥2). Patients with >1 exacerbation leading to hospital admission in the last 1 year were assigned to group C or D. Group C patients were those with mMRC score lower than 1, while those with mMRC score ≥ 2 cases belonged to group D.

### 2.3. Statistical analysis

Statistical analysis was performed using Excel and SPSS Statistics 26.0 (IBM SPSS, Chicago, IL). Measurement data, such as age, were expressed in mean differences ± standard deviation, and compared by the independent sample *t*-test if they were normally distributed. For those who did not conform to normal distribution, nonparametric Mann–Whitney *U* test was applied. The enumeration data, which was displayed by the number of cases (percentage), were analyzed by the *χ*^2^ test between the 2 groups. If the value of expected cases in 1 cell was ≥1 but <5, we adopted continuity-adjusted formula for chi-square test. Fisher Exact test was used if a cell had few expected cases (i.e.,<1) in the table. A 2-tailed *P* < .05 was considered to indicate statistical significance. The pie charts were completed by SPSS Statistics 26.0 according to the number of cases in different groups.

## 3. Results

### 3.1. Population characteristics

A total of 212 COPD patients who had not been vaccinated were recruited in our study, including 164 cases willing to accept the COVID-19 vaccination and 48 patients hesitant about vaccination. They were defined as acceptance group and hesitant group respectively. Table 1 compared the demographic and clinical details of the study populations. The mean age of acceptance group was 63.85 ± 0.72 years, having no significant difference from that of hesitant group (62.48 ± 0.53 years), with a *P* > .05. Men accounted for 75.0% and 79.2% in 2 groups respectively, and there was no statistical significance between 2 groups (*P* > .05). As showed in Table 1, compared with their counterparts, patients in the hesitant group had a higher probability to suffer from more comorbidities, including hypertension (62.5% vs 45.1%), coronary heart disease (56.3% vs 37.2%) and cancers diagnosed within 3 years (8.3% vs 0.0%). In particular, cases with >1 comorbidity had a lower willingness to be vaccinated than those patients having no comorbidities, or only 1 comorbidity. Besides that, the population studied treated with 2 bronchodilators accounted for 37.5% in the hesitant group, which was more than those in the acceptance group (6.1%), with *P* < .05. We have not found any significant differences between 2 groups at smoking status, location, highest education level, and marital status of the participants, according to our current data.

**Table 1:** Comparison of demographic characteristics of the acceptant group and hesitant group.

Characteristics	Acceptance group (n = 164)	Hesitant group (n = 48)	*P* value
	N %	N %	
Age (means ± SD, year)	63.85 ± 0.72	62.48 ± 0.53	>.05
Gender			>.05
Male	123 (75.0%)	38 (79.2%)	
Female	41 (25.0%)	10 (20.8%)	
Marital status			>.05
Married	111 (67.7%)	42 (87.5%)	
Divorced or widowed	53 (32.3%)	6 (12.5%)	
Highest education level			>.05
Senior high school and above	56 (34.1%)	11 (22.9%)	
Junior high school and below	108 (65.9%)	37 (77.1%)	
Average monthly income (CNY¥)			>.05
>6000	22 (13.4%)	4 (8.3%)	
3000–6000	60 (36.6%)	20 (41.7%)	
<3000	82 (50.0%)	24 (50.0%)	
Location			>.05
Urban	79 (48.2%)	21 (43.8%)	
Rural	85 (51.8%)	27 (56.2%)	
Smoking status			>.05
Ever	116 (70.7%)	36 (75.0%)	
Never	48 (29.3%)	12 (25.0%)	
Personal history or close acquaintances of COVID-19			>.05
Yes	5 (3.1%)	2 (4.2%)	
No	159 (96.9%)	46 (95.8%)	
Comorbidities			
Hypertension	74 (45.1%)	30 (62.5%)	<.05*
Coronary heart disease	61 (37.2%)	27 (56.3%)	<.05*
Type 2 diabetes	26 (15.9%)	8 (16.7%)	>.05
Cancer (>3 years)	3 (1.8%)	0 (0.0%)	>.05
Cancer (<3 years)	0 (0.0%)	4 (8.3%)	<.05*
Other diseases	39 (23.8%)	12 (25.0%)	>.05
Number of comorbidities			<.01*
>1	31 (18.9%)	29 (60.4%)	
≤1	133 (81.1%)	19 (39.6%)	
Vaccinated against pneumonia in the past year			>.05
Yes	61 (37.2%)	13 (27.1%)	
No	103 (62.8%)	35 (72.9%)	
Current treatment			
LABA + ICS	143 (87.2%)	30 (62.5%)	>.05
LAMA	98 (59.8%)	26 (54.2%)	>.05
LAMA + LABA	10 (6.1%)	18 (37.5%)	<.05*
LAMA + LABA + ICS	0 (0.0%)	2 (4.2%)	>.05
Theophylline	37 (22.6%)	9 (18.8%)	>.05

COVID-19 = coronavirus disease 2019, ICS = inhaled corticosteroid, LABA = long-term β2 receptor agonist, LAMA = long-acting anticholinergic.

### 3.2. Participants’ attributes toward COVID-19 vaccine

Table 2 gave us information about the concerns and motivations for COVID-19 vaccination of the 212 participants. The most common reason motivating all the 212 patients with COPD to accept vaccination was endorsement from world or national authorities (both 100.0% in 2 groups). Other contributing factors for most patients (>80%) included free vaccination, safety and effectiveness of the vaccine, and recipients they knew have no adverse reactions. Remarkably, compared with those in acceptant group (only 37.8%), patients who were concerned about the recommendations from their doctor in charge in the hesitant group were more, taking up a percentage of 75.0%. This tendency could also be seen from the concerns the participants showed about the COVID-19 vaccine. A majority of patients in the hesitant group (93.8%) worried that their treating physician would not recommend them to accept the vaccination, while, this proportion in the other group was 0.0%. This reason was the most obvious obstacle for them to accept vaccination, compared with other factors, such as distrust of vaccine safety (22.9%), and the belief that vaccination is unnecessary due to virus mutation (8.3%).

**Table 2:** Motivations and concerns regarding COVID-19 vaccination among patients with chronic obstructive pulmonary disease (COPD).

Motivations and concerns	Acceptant group (n = 164) N %	Hesitant group (n = 48) N %	*P* value
What would motivate you to be vaccinated against COVID-19?			
My doctor in charge recommended me to be vaccinated.	62 (37.8%)	36 (75.0%)	<.01*
I believe it is safe and effective	151 (92.1%)	45 (93.8%)	>.05
If someone close to me who receives it does not experience adverse reactions	160 (97.6%)	40 (83.3%)	>.05
Mandatory vaccination	12 (7.3%)	7 (14.6%)	>.05
Free vaccination	160 (97.6%)	45 (93.8%)	>.05
World or national authorities have endorsed it	164 (100.0%)	48 (100.0%)	>.05
COVID-19 causes death	57 (34.8%)	14 (29.2%)	>.05
Patients still have long-term sequelae after recovery	96 (58.5%)	35 (72.9%)	>.05
What concerns do you have about the COVID-19 vaccine?			
Distrust of vaccine safety	13 (7.9%)	11 (22.9%)	<.05*
Distrust of vaccine effectiveness	2 (12.2%)	1 (2.1%)	>.05
Distrust of domestic vaccine	0 (0.0%)	0 (0.0%)	>.05
Treating physician has not recommended it	0 (0.0%)	45 (93.8%)	<.01*
Believe that it is unnecessary to receive the vaccine due to virus mutation	18 (11.0%)	4 (8.3%)	>.05

COVID-19 = coronavirus disease 2019.

### 3.3. COVID-19 vaccination information in different COPD groups

**Figure 1.**
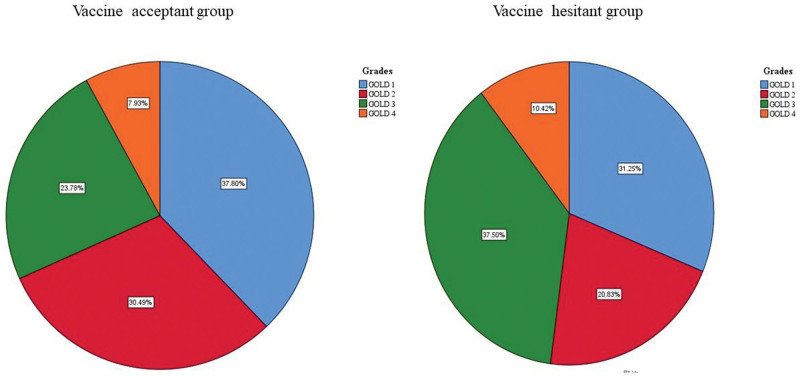
COVID-19 vaccination ratio at different GOLD grades of vaccine-acceptant group and vaccine-hesitant group. COVID-19 = coronavirus disease 2019, GOLD = Global Initiative for Chronic Obstructive Lung Disease.

**Figure 2.**
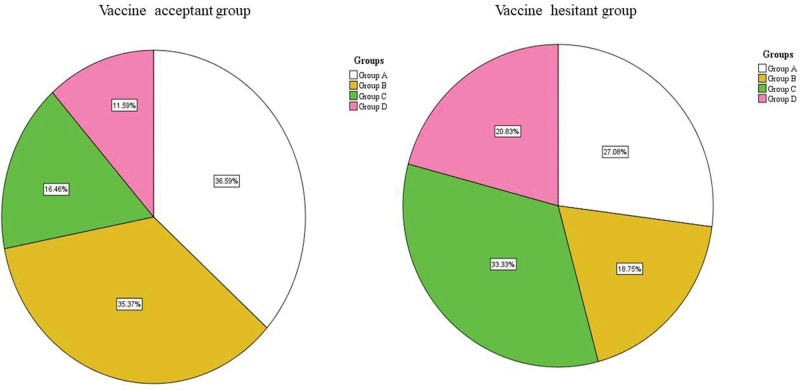
COVID-19 vaccination ratio in different COPD groups (Group A, B, C, D). COPD = chronic obstructive pulmonary disease, COVID-19 = coronavirus disease 2019.

All the participants in 2 groups were further classified into 4 grades (GOLD 1, GOLD 2, GOLD 3, GOLD 4) and 4 groups (Group A, Group B, Group C, Group D) according to airflow limitation severity, mMRC score, as well as the risk of exacerbation in last 1 year. As shown in Table 3 and Figure 1, although there was no statistical significance, the trend that patients with COPD in the hesitant group had more severe airway limitation still existed. GOLD 3 cases took a percentage of 37.5% and 23.8% in the hesitant group and acceptant group respectively. This difference in GOLD 4 was consistent but with a small margin (10.4% vs 7.9%). Table 4 and Figure 2 illustrate the number of cases who were willing or unwilling to be vaccinated immediately in various populations grouped by exacerbation risk and mMRC score. As shown, for the acceptant group patients, the leading part was Group A, accounting for 36.6%, followed by Group B (35.4%), Group C (16.5%), and Group D (11.6%). Regarding the hesitant group, the dominant part was group C patients (33.3%). Likewise, the group D cases, with a proportion of 20.8%, were more than their counterparts (*P* < .05). Statistically significant difference in group B cases was also observed, and the proportion of them in the hesitant group was only 18.8%. Generally, more patients in the hesitant group had higher mMRC scores or more frequent acute exacerbation.

**Table 3:** COVID-19 vaccination numbers at different GOLD grades of 2 groups.

GOLD grades	Acceptant group (n = 164) N %	Hesitant group (n = 48) N %	*P* value
GOLD 1	62 (37.8%)	15 (31.3%)	>.05
GOLD 2	50 (30.5%)	10 (20.8%)	>.05
GOLD 3	39 (23.8%)	18 (37.5%)	>.05
GOLD 4	13 (7.9%)	5 (10.4%)	>.05

COVID-19 = coronavirus disease 2019, GOLD = Global Initiative for Chronic Obstructive Lung Disease.

**Table 4:** COVID-19 vaccination numbers in Group A to D.

Groups	Acceptant group (n = 164) N %	Hesitant group (n = 48) N %	*P* value
A	60 (36.6%)	13 (27.1%)	>.05
B	58 (35.4%)	9 (18.8%)	<.05*
C	27 (16.5%)	16 (33.3%)	<.05*
D	19 (11.6%)	10 (20.8%)	<.05*

COVID-19 = coronavirus disease 2019.

## 4. Discussion

In this study, we investigated the attitudes of 212 patients with COPD toward COVID-19 vaccine. Through comparing the clinical features, motivations, and concerns they had for vaccination, we drew the conclusion that patients who were hesitant to be vaccinated instantly tended to have more comorbidities, like hypertension, coronary heart disease, recent cancers, and higher mMRC score, or more frequent acute exacerbation. Additionally, our data demonstrated that the main motivational factors for all participants were authorities-endorsed vaccine, free vaccination, and no obvious adverse reactions. For the hesitant group, no recommendation from the treating physician was the biggest obstacle for them to accept vaccination. To the best of our knowledge, this is the first study conducted to measure the acceptability of the anti-SARS-CoV-2 vaccination in this specific population. Previous studies on breast cancer patients showed 183 (34%) of 540 participants were hesitant to be vaccinated and 357 (66%) were willing to be vaccinated immediately. By comparison, participants in the acceptance group took a higher proportion in our study. The main 3 reasons motivated vaccine-acceptance patients were to prevent COVID-19, to take care of their relatives, and an auto-perceived social responsibility.^[11]^

COVID-19, which is caused by SARS-CoV-2, has raised public concern globally since it is characterized as a pandemic by the WHO.^[12]^ Previous clinical and epidemiological data suggested that some comorbidities increase the SARS-CoV-2 infection risk with worse lung injury and death.^[13]^ For example, in patients suffering from hypertension, angiotensin-converting enzyme 2 receptor is easier to have an upregulated expression due to the frequently using of angiotensin-converting enzyme 2 inhibitors and angiotensin receptor blockers. These overexpressed receptor cells in the lung result in high susceptibility to COVID-19, more chances of severe lung injury, and respiratory failure.^[14]^ As for CVD, in a previous study, it was proved to have a strong relationship with SARS (8%) and Middle East Respiratory Syndrome (30%). Previous research in Wuhan noted that as high as 17% of the COVID-19 nonsurvivors had CVD, while the exact mechanism is not precise.^[15]^ COPD is associated with COVID-19 infection and also with a high case fatality rate.^[16]^ The main reason is COPD is often accompanied by continual mucus production, respiratory corticosteroid use, weak immunity, structural damage, and microbiome imbalance.^[17]^ Related studies demonstrated that COPD could be seen in 50 to 52.3% of the total ICU-admitted COVID-19 cases, leading to high mortality among these patients.^[18]^ However, even more comorbidities mean high mortality to COVID-19 sufferers, according to our data, these patients were not willing to be vaccinated immediately because of worrying about no recommendation from the treating physician and distrust of vaccine safety. Actually, only <0.06% of the individuals who have accepted vaccination had systemic side effects.^[19]^ The most common symptoms after being vaccinated, according to a survey involved with 239.97 million doses of COVID-19 vaccination, were headache (0.01%) and fever (0.01%), and the incidence rate of serious side effects including death (0.002%) was extremely low.^[19]^ In special populations, take older people for example, the data from Phase II trials suggested mild to moderate adverse events in well older people compared to younger participants. However, older people with comorbidities and frailty have been largely excluded in Phase II and III trials of Pfizer and Moderna vaccine, thus there are no sufficient published data on safety in this group.^[20]^

Several types of COVID-19 vaccines worldwide have been approved by the WHO, including live attenuated vaccines, mRNA vaccines, and inactivated SARS-CoV-2 vaccines.^[21]^ Compared with the first 2 vaccines, SARS-CoV-2 vaccines such as CoronaVac (SinoVac Biotech, Beijing, China) generate a weaker immune response but rare side effects.^[22,23]^ Some researchers even suggested that acute urticaria alone, which belongs to immediate allergic reactions and is recommended not to receive the same mRNA and adenoviral vector COVID-19 vaccines, should not be a contraindication for the second dose of CoronaVac.^[24]^ Treating physicians, whose opinions are of great importance for hesitant participants, could recommend their patients with COPD to accept inactivated SARS-CoV-2 vaccines if they are extremely worried about their severe clinical symptoms.

In conclusion, our study identified the COPD patients’ attitudes toward COVID-19 vaccine. It may provide useful guidance for making intervention measures to enhance COPD patients’ acceptance of a new COVID-19 vaccine.

## Author contributions

**Data curation:** Lele Bai.

**Visualization:** Ning Wang.

**Writing – original draft:** Ting Wang.

**Writing – review & editing:** Yang Bai, Ning Wang.
